# Dyke–Davidoff–Masson syndrome: A rare case report

**Published:** 2014-10-06

**Authors:** Deepak Jain, Hari Krishan Aggarwal, Shivraj Goyal, Ansul Mittal

**Affiliations:** Department of Medicine, Pandit Bhagwat Dayal Sharma Post Graduate Institute of Medical Sciences, Rohtak, Haryana, India

**Keywords:** Dyke–Davidoff–Masson Syndrome, Cerebral Hypoplasia, Recurrent Seizure

We hereby report a 30-year-old young male who had a history of generalized tonic-clonic seizures since age of 5 years. Patient took antiepileptic drug treatment, including phenytoin and phenobarbitone and some indigenous medications. However, continued to have a seizure on and off and presented in an emergency department with intractable seizures. Patient had history of weakness left half of the body for last 8 years, non-progressive in nature. His mother revealed history of developmental delay including gross motor, fine motor, language, perceptive cognitive, social and personal delay. There was no history of similar illness in any other sibling or family member.

On examination, patient was conscious with an intelligence quotient of 60 indicating mild mental retardation. Neurological examination revealed 4/5 power in left upper and lower limb with brisk reflexes. Left planter was extensor. There was no neck rigidity, any sensory deficit, cranial nerve or bowel bladder involvement.

All routine biochemical investigations including liver and renal function were within normal limits. Random blood sugar, serum electrolytes including calcium levels were within normal range. Computed tomography scan showed hyperpneumatization of frontal sinus, thickened calvarial bone with hyperpneumatization of mastoid air cells (Figure 1). Magnetic resonance imaging (MRI) brain showed loss of volume of right cerebral hemisphere with gliosis and prominent right lateral ventricle and cortical sulci associated with ipsilateral calvarial thickening. Diffuse cerebellar atrophy was also noted. There was no evidence of any focus of restricted diffusion to suggest acute infarct (Figure 2). Twenty-three channel electroencephalography recordings under sedation showed a poorly organized background activity of bursts of generalized spike and wave discharged admixed with low and high voltage slow waveforms. Based on characteristic radiological finding patient was diagnosed as a case of Dyke–Davidoff–Masson syndrome (DDMS) and was treated with valproic acid and levetiracetam. Patient was controlled on medication for next 4 months but then, unfortunately, lost to follow-up.

The DDMS first reported in 1933, is a rare condition, and refers to atrophy or hypoplasia of one cerebral hemisphere (hemiatrophy).^1^ It is characterized clinically by variable degrees of facial asymmetry, recurrent seizures, contralateral hemiparesis, mental retardation, speech and language disorders along with various learning disabilities. Seizures can be focal or generalized. Occasionally, psychiatric manifestations have also been reported. Both sexes and hemispheres may be affected with a slight preponderance for male sex and left hemisphere.^2^

The cause is cerebral insult, which may occur in the prenatal, perinatal or postnatal period. Prenatal causes are congenital malformation, infection and vascular insult while, in the perinatal period birth trauma, anoxia, hypoxia and intracranial hemorrhage are most common causes. Trauma, tumor, infection and prolonged febrile seizures account for postnatal causes.^3^

**Figure 1:**
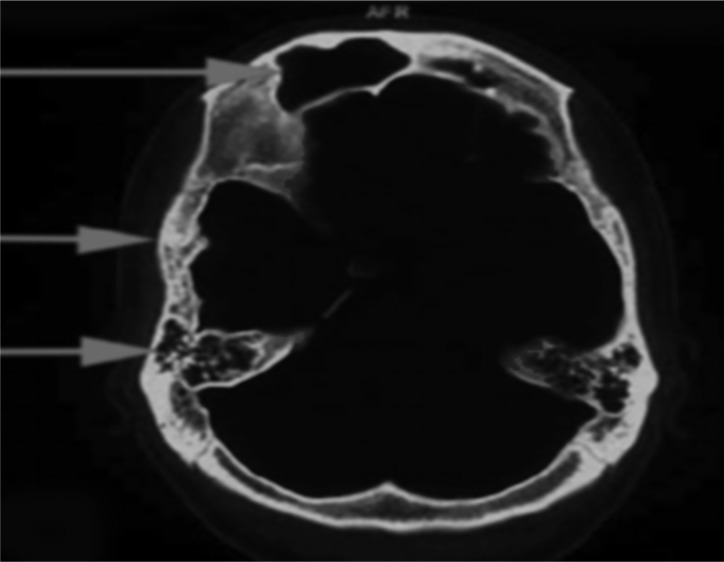
Computed tomography scan showing hyperpneumatization of frontal sinus, thickened calvarial bone with hyperpneumatisation of mastoid air cells

Since primary cerebral atrophy is actually a lack of cerebral development rather than atrophy the terms cerebral hemi-hypoplasia or unilateral cerebral hypoplasia would be more appropriate.^4^ The brain reaches half of its adult size during the 1^st^ year of life and reaches three-fourths of that size by the end of 3^rd^ year. As it enlarges, the brain presses outward on the bony tables and is partly responsible for the gradual enlargement and general shape of an adult's head. When the brain fails to grow, other structures tend to direct their growth inward, thus accounting for the enlargement of the frontal sinus with increased width of the diploic space and the elevations of the greater wing of sphenoid and the petrous ridge on the affected side.^2^

The radiological features are unilateral loss of cerebral volume and associated compensatory bone alterations in the calvarium, such as thickening, hyperpneumatization of the paranasal sinuses and mastoid cells and elevation of the petrous ridge. MR represents the imaging procedure of choice to assess etiology and extent of cerebral parenchymal involvement.^5^ Shen et al. depicted three MRI patterns of cerebral hemiatrophy: MRI pattern I corresponds to diffuse cortical and subcortical atrophy; pattern II corresponds to diffuse cortical atrophy coupled with porencephalic cysts; and pattern III corresponds to previous infarction with gliosis in the middle cerebral artery territory.^5^

This condition is to be differentiated from Basal ganglia germinoma, Sturge Weber syndrome, Silver-Russel syndrome, Fishman syndrome, and Rasmussen encephalitis .In our case, classical radiological findings consistent with the DDMS pattern III were present in the background of clinical manifestations in the form of recurrent seizures and hemiparesis. The atrophied cerebral hemisphere with prominent sulcal spaces points that the vascular insult occurred after birth or after the end of sulcation.

The treatment focuses on control of the seizures with suitable anticonvulsants regimen. Along with drugs, physiotherapy, occupational therapy, and speech therapy play a significant role in the long-term management. Prognosis is better if the onset of hemiparesis is after 2 years of age and in the absence of prolonged or recurrent seizure. Hemispherectomy is the treatment of choice for children with intractable disabling seizures and hemiplegia with a success rate of 85% in selected cases.^4^

**Figure 2:**
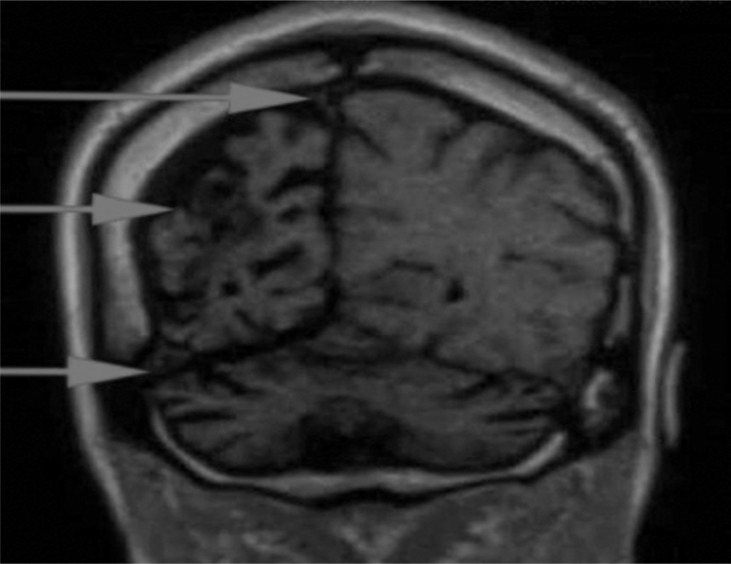
Magnetic resonance imaging scan revealing ipsilateral displacement of falx cerebri, cerebral atrophy and elevation of petrous ridge

## Notes

**How to cite this article: **Jain D, Aggarwal HK, Goyal S, Mittal A. Dyke–Davidoff–Masson syndrome: A rare case report. Iran J Neurol 2014; 13(4): 255-6.
